# Src/NF-κB-Targeted Anti-Inflammatory Effects of *Potentilla glabra* var. Mandshurica (Maxim.) Hand.-Mazz. Ethanol Extract

**DOI:** 10.3390/biom10040648

**Published:** 2020-04-22

**Authors:** Haeyeop Kim, Kon Kuk Shin, Han Gyung Kim, Minkyeong Jo, Jin Kyeong Kim, Jong Sub Lee, Eui Su Choung, Wan Yi Li, Sang Woo Lee, Kyung-Hee Kim, Byong Chul Yoo, Jae Youl Cho

**Affiliations:** 1 Department of Integrative Biotechnology, Sungkyunkwan University, Suwon 16419, Korea; rlagoduq7283@naver.com (H.K.); shuka337@naver.com (K.K.S.); hanks523@skku.edu (H.G.K.); whalsrud1017@naver.com (M.J.); rosekim95@naver.com (J.K.K.); 2 Research Institute of Biomolecule Control and Biomedical Institute for Convergence at SKKU (BICS), Sungkyunkwan University, Suwon 16419, Korea; 3 DanjoungBio Co., Ltd., Wonju 26303, Korea; js.lee@danjoungbio.com (J.S.L.); esavella@hanmail.net (E.S.C.); 4 Institute of Medicinal Plants, Yunnan Academy of Agricultural Sciences, Kunming 650224, China; wyli2012@126.com; 5 International Biological Material Research Center, Korea Research Institute of Bioscience and Biotechnology, Daejeon 34141, Korea; ethnolee@kribb.re.kr; 6 Biomarker Branch, Research Institute, National Cancer Center, Goyang 10408, Korea; kyunghee@ncc.re.kr

**Keywords:** Pg-EE, anti-inflammatory effect, Src, gastritis

## Abstract

Inflammation is a complex protective response of body tissues to harmful stimuli. Acute inflammation can progress to chronic inflammation, which can lead to severe disease. Therefore, this research focuses on the development of anti-inflammatory drugs, and natural extracts have been explored as potential agents. No study has yet examined the inflammation-associated pharmacological activity of *Potentilla glabra* Var. mandshurica (Maxim.) Hand.-Mazz ethanol extract (Pg-EE). To examine the mechanisms by which Pg-EE exerts anti-inflammatory effects, we studied its activities in lipopolysaccharide (LPS)-treated murine macrophage RAW264.7 cells and an HCl/EtOH-induced gastritis model. LPS-triggered nitric oxide (NO) release and mRNA levels of inducible nitric oxide synthase (iNOS), tumor necrosis factor-alpha (TNF-α), interleukin-6 (IL-6), and interleukin-1 beta (IL-1β) in RAW264.7 cells were suppressed by Pg-EE in a dose-dependent manner. Using a luciferase assay and western blot assay, we found that the NF-κB pathway was inhibited by Pg-EE, particularly by the decreased level of phosphorylated proteins of nuclear factor kappa-light-chain-enhancer of activated B cells (NF-κB) subunits (p65 and p50), inhibitor of kappa B alpha (IκBα), p85, and Src. Using an overexpression strategy, cellular thermal shift assay, and immunoprecipitation analysis, we determined that the anti-inflammatory effect of Pg-EE was mediated by the inhibition of Src. Pg-EE further showed anti-inflammatory effects in vivo in the HCl/EtOH-induced gastritis mouse model. In conclusion, Pg-EE exerts anti-inflammatory activities by targeting Src in the NF-κB pathway, and these results suggest that Pg-EE could be used as an anti-inflammatory herbal medicine.

## 1. Introduction

Inflammation is a type of immune response to stimuli such as a pathogen and injury [1,2]. Various immune cells are involved in the inflammation response, and they try to remove the stimuli to relieve inflammatory symptoms, such as pain, redness, swelling, heat, and loss of function [3]. Therefore, inflammation can protect the body of an organism; however, if this acute inflammation becomes uncontrolled over a prolonged period, it can develop into chronic inflammation [4], which can cause severe diseases, such as autoimmune diseases, rheumatoid arthritis, asthma, and cancers [5]. The immune system can be divided into two types: innate immunity and adaptive immunity. Innate immunity is a non-specific immune response and mostly occurs at early infection. Macrophages, epithelial cells, and natural killer cells are involved in this type of immunity [6]. The non-specific response of innate immunity involves the interaction of pattern recognition receptors (PRRs) and pathogen-associated molecular patterns (PAMPs). PRRs are receptors that recognize PAMPs, which are present according to the type of pathogen [7].

Toll-like receptors (TLRs) are a type of PRR, and each TLR dimerizes and binds with a specific PAMP. [8] TLR4 interacts with lipopolysaccharide (LPS), TLR3 interacts with Poly(I:C), and TLR1/2 heterodimers interact with Pam3CSK4 [9,10]. Upon PRR binding to PAMPs, intracellular signaling is activated. Among these bindings, the interaction between TLR4 and LPS is the most powerful and common in activating inflammatory responses in macrophages [11]. TLR signaling transduces inflammatory signal cascades via two major adaptor molecules: Toll/interleukin-1 receptor-domain-containing adapter-inducing interferon-β (TRIF) and myeloid differentiation response gene 88 (MyD88) [12]. The activation of protein tyrosine kinases Syk and Src, phosphoinositide 3-kinase (PI3K), AKT, and IκB kinase (IKK) induces the nuclear translocation of transcriptional factors, such as nuclear factor kappa-light-chain-enhancer of activated B cells (NF-κB) and activator protein-1 (AP-1) [9,13,14].

The activation of NF-κB and AP-1 transcription factors leads to the upregulation of inflammatory genes, including inducible nitric oxide synthase (iNOS) and cyclooxygenase-2 (COX-2) [15], and the production of various cytokines, such as tumor necrosis factor-alpha (TNF-α), interleukin-1 beta (IL-1β), interleukin-6 (IL-6), interleukin-12 (IL-12), and interferons (IFNs) from activated macrophages [16,17]. These cytokines promote the initiation and amplification of inflammation and injury in the body [18]. Finally, inflammatory mediators, such as nitric oxide (NO), which is produced by iNOS, and prostaglandin E_2_ (PGE_2_), which mediates heat, swelling, vasodilation, and inflammation, are produced [19]. When these inflammatory pathways are overactive in the body, inflammation is induced and leads to severe diseases. Therefore, to suppress these pathways, it is important to relieve the inflammation and this is the goal of this research.

Here, we explored the use of traditional herbal remedies for anti-inflammatory effects and safety [13,20,21]. Various species of *Potentilla* have long been used in traditional medicine in Asia, Europe, and North America [22]. Extracts from *Potentilla* species exhibit antioxidant, antimicrobial, anti-inflammatory, and anti-ulcerogenic properties [23,24]. Currently, however, no studies have examined the potential anti-inflammatory effects of *Potentilla glabra* var. mandshurica (Maxim.) Hand.-Mazz. (Pg-EE) and its molecular mechanism, although *Potentilla* species have been ethnopharmacologically used for a long time in many countries. In this study, we focused on exploring the anti-inflammatory efficacy of *Potentilla glabra* at the cellular, molecular, and animal-model levels. For this, we employed LPS-induced macrophages and an HCl/EtOH-induced gastritis mouse model and identified a molecular pharmacological target by using an overexpression strategy.

## 2. Materials and Methods

### 2.1. Materials

A 95% ethanol extract of Pg-EE was obtained from the International Biological Material Research Center (Daejeon, Korea). LPS, (3-4,5-dimethylthiazol-2-yl)-2,5-diphenyl-tetrazolium bromide (MTT), N(G)-Nitro-l-arginine methyl ester (l-NAME), ranitidine, pam3CSK4 (Pam3), Poly(I:C), quercetin, polyethylene imidazole (PEI), and sodium dodecyl sulfate (SDS) were purchased from Sigma Chemical Co. (St. Louis, MO, USA). Roswell Park Memorial Institute (RPMI) 1640, fetal bovine serum (FBS), Dulbecco’s Modified Eagle’s medium (DMEM), phosphate buffered saline (PBS), and TRIzol reagent were purchased from GIBCO (Grand Island, NY, USA). RAW264.7 cells (ATCC number TIB-71) and HEK293T cells (ATCC number CRL-1573) were purchased from the American Type Culture Collection (ATCC) (Rockville, MD, USA). Antibodies specific for phosphorylated and total forms of p65, p50, inhibitor of kappa B alpha (IκBα), Src, p85/PI3K, AKT, and β-actin were purchased from Cell Signaling Technology (Beverly, MA, USA).

### 2.2. Animals

Institute of Cancer Research (ICR) mice (male, 6–8 weeks old) were purchased from Daehan Biolink (Osong, Korea) and housed in plastic cages under standard conditions. Water and feed (Samyang, Daejeon, Korea) were given ad libitum. All studies were conducted according to the guidelines of the Institutional Animal Care and Use Committee at Sungkyunkwan University (Suwon, Korea; approval ID: SKKUIACUC2019-07-12-1).

### 2.3. Cell Culture

RAW264.7 cells and HEK293T cells were cultured in RPMI 1640 medium with 10% heat-inactivated FBS and antibiotics (penicillin and streptomycin) at 37 °C under 5% CO_2_ and DMEM medium with 5% heat-inactivated FBS and antibiotics (penicillin and streptomycin) at 37 °C under 5% CO_2_, respectively.

### 2.4. Cell Viability Test

The cytotoxicity of Pg-EE for 24 and 48 h in RAW264.7 cells (1 × 10^6^ cells/mL) and HEK293T cells (2 × 10^5^ cells/mL) was measured by MTT assays. Cells were treated with Pg-EE for various times; next, 10 μL of MTT solution (10 µg/mL in PBS, pH 7.4) was added, and the cells were cultured for 3 h. The assay was stopped by adding 15% sodium dodecyl sulfate to each well to dissolve the formazan [25]. Absorbance at 570 nm (OD570–630) was measured using a Synergy HT Multi-Mode Microplate Reader (BioTek Instruments, Inc., headquartered in Winooski, VT, USA) [26].

### 2.5. Nitric Oxide (NO) Assay

RAW264.7 cells (1 × 10^6^ cells/mL) were plated in 96-well plates and pretreated with Pg-EE (0–150 µg/mL) or L-NAME (0–2 mM) for 30 min. Cells were then further incubated with inflammatory stimuli (LPS (1 µg/mL), Pam3CSK4 (20 µg/mL), or Poly(I:C) (200 µg/mL)) for 24 h. Next, 100 µL of supernatant was obtained and mixed with 100 μL of Griess reagent, as reported previously [27]. The absorbance was measured at 540 nm using a multi-reader. The final concentration of NO was calculated using an NO standard.

### 2.6. High-Performance Liquid Chromatography (HPLC)

To verify the phytochemical characteristics of Pg-EE, HPLC was conducted as reported previously [28,29]. Quercetin, naringenin, kaempferol, silibinin, genistein, and apigenin were used as standard components.

### 2.7. mRNA Analysis by a Quantitative Reverse Transcriptase-Polymerase Chain Reaction

RAW264.7 cells (1 × 10^6^ cells/mL) were treated with Pg-EE (0–150 µg/mL) in advance and induction was performed with LPS (1 µg/mL) after 30 min. After 6 h of induction, the total RNA was extracted using TRIzol reagent. cDNA was synthesized from 1 μg of total RNA using MuLV reverse transcriptase, as described previously [30]. The total RNA and synthesized cDNA were also obtained from stomach tissues of HCl/EtOH-induced gastritis mice. The primer sequences are listed in Table 1.

### 2.8. Plasmid Transfection and Luciferase Reporter Gene Activity Assay

HEK293T cells (2.5 × 10^5^ cells/mL) were seeded in 24-well plates and transfected with NF-κB-Luc or a plasmid encoding β-galactosidase (0.25 μg/mL) using the polyethylenimine (PEI) method, as described previously [31]. After 24 h, the transfected cells were treated with Pg-EE (0–150 μg/mL) and with or without LPS. The cells were harvested and lysed by freezing at −70 °C for at least 3 h. The luciferase reporter activity was determined by measuring the luminescence using a luminometer, which was then normalized to that of β-galactosidase activity [32].

### 2.9. Western Blot Analysis and Immunoprecipitation

RAW264.7 cells (1 × 10^6^ cells/mL) were pretreated with Pg-EE (150 μg/mL) for 30 min and subsequently, LPS (1 μg/mL) induction was processed for a designated time. HEK293T cells were transfected with a specific gene, incubated for 24 h, and then processed with Pg-EE for another 24 h. Whole cell lysates and stomach lysates of gastritis mice were prepared for western blot analysis, as previously described [33,34]. Western blot analyses using whole cell lysates and stomach lysates were conducted as previously described using antibodies specific for each target protein [33].

For immunoprecipitation, cell lysates containing equal amounts of protein (1000 ng) from RAW264.7 cells treated with or without LPS (1 μg/mL) for 5 min were prepared. Pre-cleared samples were incubated with 0.4 μL antibodies to IgG or 3 μL antibodies to Src overnight at 4 °C. Immune complexes were mixed with 50 μL of protein A-coupled Sepharose beads (50% *v*/*v*) and rotated for 4 h at 4 °C, after which they were washed with a buffer (50 mM Tris-HCl pH 7.5, 20 mM NaF, 25 mM β-glycerol phosphate pH 7.5, 120 mM NaCl, 2% NP-40, phosphatase/protease inhibitors). Next, 70 µL of 1x sample buffer (25% glycerol, 2% SDS, 60 mM Tris-HCl pH 6.8, 5% 2-mercaptoethanol, 0.1% bromophenol blue) was added, and samples were heated at 90 °C for 3 min. The supernatants were then examined by western blot analysis [35].

### 2.10. Cellular Thermal Shift Assay (CETSA)

HEK293T cells were transfected by plasmids expressing Src or Src domain deletion genes and treated with DMSO or Pg-EE (150 µg/mL) for 24 h. After treatment, the cells were isolated and resuspended in PBS. Then, the suspended cells were separated into seven PCR tubes in a volume of 100 µL with the same number of cells. Each PCR tube was heated for 3 min at a temperature gradient from 49 to 61 °C, and then cooled to 25 °C for 3 min. We performed three rounds of freezing-thawing using liquid nitrogen and room-temperature water. The samples were transferred into each Eppendorf tube and centrifuged at 12,000 rpm for 30 min. Protein samples were examined by western blot analysis.

### 2.11. HCl/EtOH-Induced Gastritis Model

ICR mice (four mice per group) were injected with HCl/60% EtOH to generate the acute gastritis model [36]. Fasted ICR mice were orally injected with Pg-EE (100 or 150 mg/kg) or ranitidine (40 mg/kg) twice per day for 2 days. At 8 h after the last oral injection, all groups, except for the control group, were orally administered 300 µL of 150 mM HCl/60% EtOH for 1 h. All groups of ICR mice were sacrificed; the stomachs were separated from the mice and washed with PBS, and then split in half to confirm the lesion.

### 2.12. Statistical Analysis

For statistical comparisons, a student’s *t*-test and one-way ANOVA were used to determine the statistical significance of the difference between values for the various experimental and control groups. Data are expressed as the mean ± standard error, and the results were obtained from at least three independent experiments performed in triplicate. A *p*-value < 0.05 was considered statistically significant.

## 3. Results

### 3.1. Pg-EE Suppressed the NO Production Level

**Figure 1:**
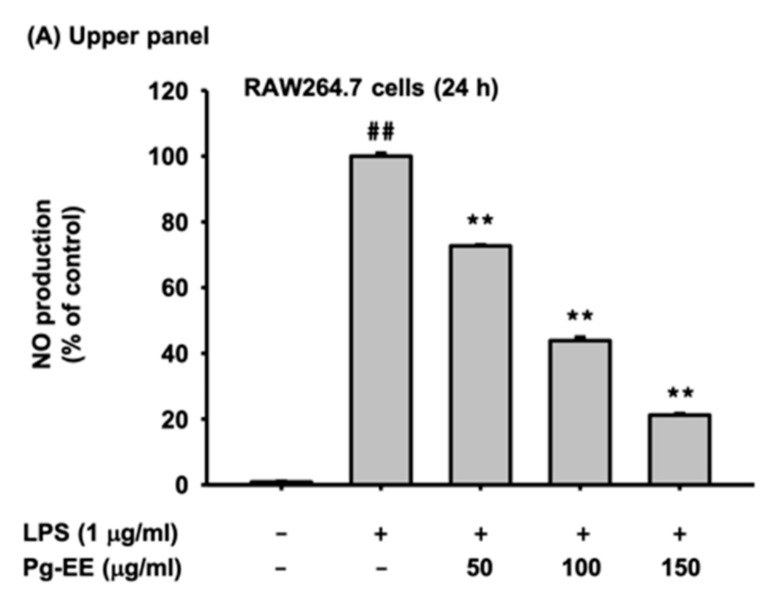

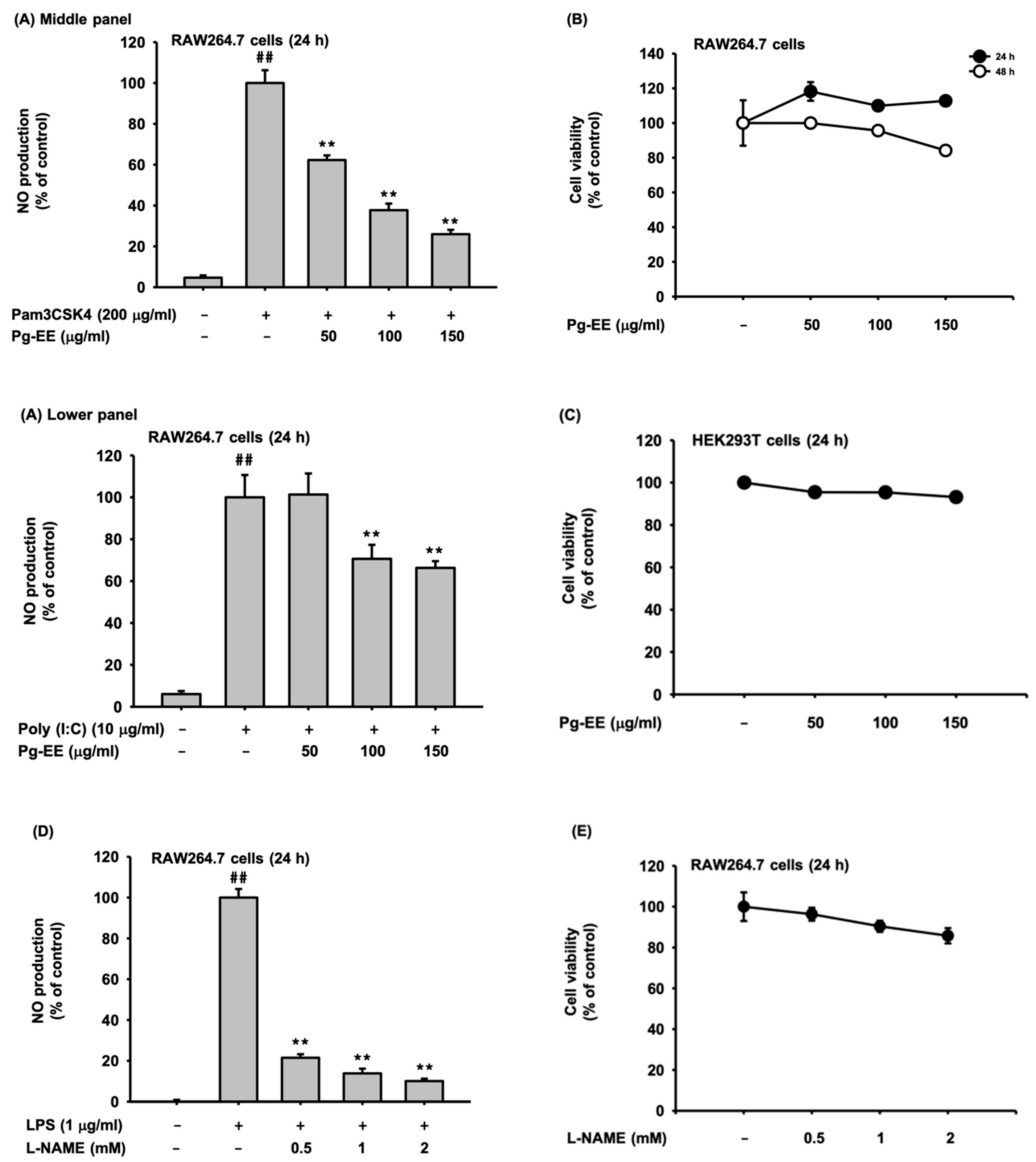

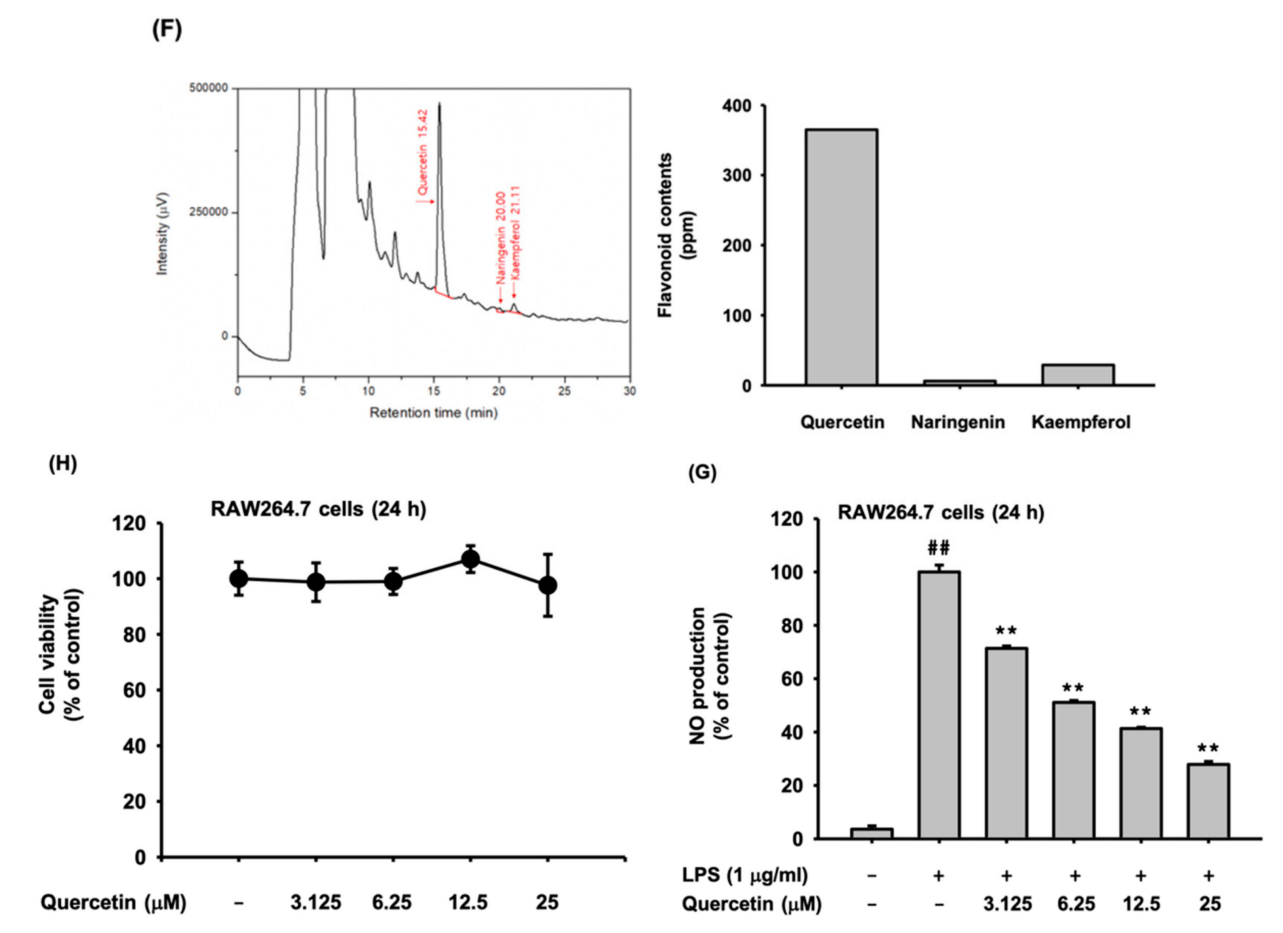
In vitro anti-inflammatory effects of *Potentilla glabra* Var. mandshurica (Maxim.) Hand.-Mazz ethanol extract (Pg-EE). (**A**) The nitric oxide (NO) production level in the culture supernatant of RAW264.7 cells treated with lipopolysaccharide (LPS) (1 μg/mL) (upper panel), Pam3CSK4 (200 μg/mL) (middle panel), or Poly(I:C) (10 μg/mL) (lower panel), with or without Pg-EE for 24 h, was analyzed with a Griess assay. (**B**,**C**) RAW264.7 cells and HEK293T cells were treated with indicated concentrations of Pg-EE (0–150 μg/mL) for 24 and 48 h and determined using the (3-4,5-dimethylthiazol-2-yl)-2,5-diphenyl-tetrazolium bromide (MTT) assay. (**D**,**E**) RAW264.7 cells were treated with various concentrations of N(G)-Nitro-l-arginine methyl ester (L-NAME) (0–2 mM) for 24 h. The NO production level in the culture supernatant of RAW264.7 cells treated with LPS was analyzed with a Griess assay, and the cell viability was detected with the MTT assay. (**F**) The contents of Pg-EE were detected by high-performance liquid chromatography (HPLC) analysis using standard flavonoids (quercetin, naringenin, kaempferol, silibinin, genistein, and apigenin). (**G**,**H**) RAW264.7 cells were treated with various concentrations of quercetin (3.125–25 µM) for 24 h. The NO production level in the culture supernatant of RAW264.7 cells treated with LPS was analyzed with a Griess assay, and the cell viability of quercetin was determined with the MTT assay. ##: *p* < 0.05 compared to the normal group, *: *p* < 0.05 and **: *p* < 0.01 compared to the control group. −: no treatment and +: treatment.

To find out the anti-inflammatory effect of Pg-EE, we first performed a nitric oxide production assay. In this study, we used RAW264.7 cells induced with TLR ligands of LPS (ligand of TLR4), Poly(I:C) (ligand of TLR3), and Pam3CSK4 (ligand of TLR1/2). The nitric oxide production level was decreased by Pg-EE dose-dependently (Figure 1A). We further examined the cell cytotoxicity of Pg-EE (50–150 µg/mL) in RAW264.7 cells and HEK293T cells by the MTT assay (Figure 1B,C). Pg-EE did not show any cell cytotoxicity in both cells up to 48 h, implying that NO inhibitory activity of Pg-EE is not derived by its non-specific cytotoxic activity.

Next, we checked the suppressive effect of L-NAME (NOS inhibitor) as a positive control by measuring the production level of NO in LPS-stimulated RAW264.7 cells (Figure 1D,E). Finally, we employed HPLC analysis to determine the active pharmacological components of Pg-EE. Through HPLC analysis, we tried to quantify what kinds of flavonoids are contained in Pg-EE, and several types of flavonoids (quercetin, naringenin, kaempferol, silibinin, genistein, and apigenin), which are well-known to have an anti-inflammatory effect, were used as the standard. Among the various flavonoids used as standard contents, Pg-EE has only three kinds of flavonoids: quercetin, naringenin, and kaempferol. The results showed that the contents of Pg-EE include 365 ppm of quercetin, 6 ppm of naringenin, and 29 ppm of kaempferol (Figure 1F). These findings indicate that the major component of Pg-EE is quercetin, which is well-known to have an anti-inflammatory effect [37]. Therefore, we examined whether quercetin can inhibit NO production. As Figure 1G shows, this flavonoid decreased NO production in a dose-dependent manner, without altering the cell viability (Figure 1G,H), implying that anti-inflammatory activity of Pg-EE could be derived by the action of quercetin.

### 3.2. Anti-Inflammatory Effects of Pg-EE at the Transcriptional Level

**Figure 2:**
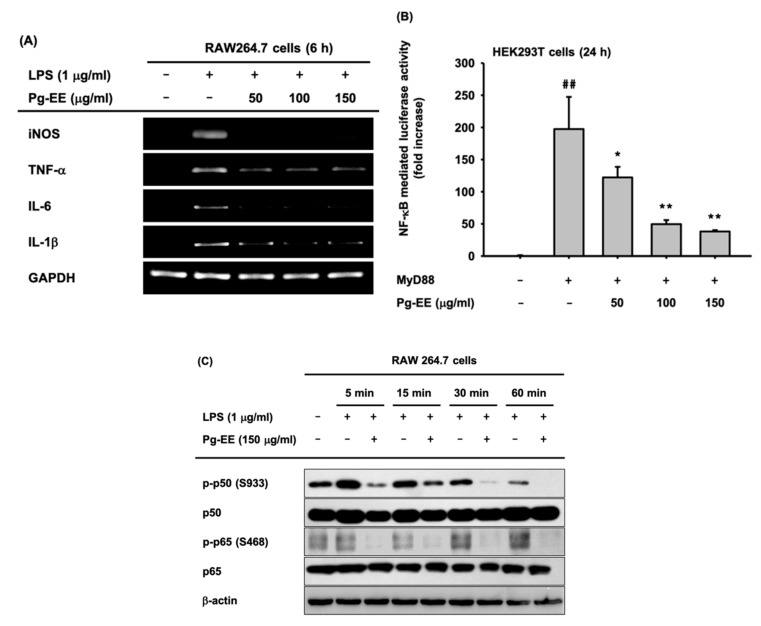
(**A**) The mRNA expression levels of inducible NO synthase (iNOS), tumor necrosis factor-alpha (TNF-α), interleukin-6 (IL-6), interleukin-1 beta (IL-1 β), and glyceraldehyde 3-phosphate dehydrogenase (GAPDH) in LPS-stimulated RAW264.7 cells treated with Pg-EE (0–150 µg/mL) were detected by RT-PCR. (**B**) The effect of Pg-EE (0–150 μg/mL) on the activation of nuclear factor kappa-light-chain-enhancer of activated B cells (NF-κB) was recognized by a luciferase reporter gene assay in HEK293T cells co-transfected with an NF-κB luciferase construct, as well as a β-gal-expressing plasmid (as a transfection control), with or without myeloid differentiation primary response 88 (MyD88). Luciferase activity was determined using a luminometer. (**C**) The total and phospho-protein levels of p50 and p65, which are NF-κB subunits, and β-actin were determined by immunoblotting analysis using whole cell lysates from LPS (1 μg/mL)-stimulated RAW264.7 cells in the presence or absence of Pg-EE (150 µg/mL). ##: *p* < 0.05 compared to the normal group, *: *p* < 0.05 and **: *p* < 0.01 compared to the control group.

Next, we checked the transcriptional level of pro-inflammatory genes by semi-quantitative RT-PCR. iNOS, TNF-α, IL-6, and IL-1β are pro-inflammatory cytokines and their gene expression levels were decreased by Pg-EE treatment dose-dependently (Figure 2A). We next examined which of the various anti-inflammatory signal pathways were blocked by Pg-EE. Since the mRNA expression levels of pro-inflammatory cytokines, such as TNF-α, IL-6, and IL-1β, are related to the NF-κB pathway [38], we expected Pg-EE to attenuate the NF-κB pathway. To examine this hypothesis, we conducted NF-κB-mediated luciferase assays. The activation of transcription factor and NF-κB were induced by treating HEK293T cells with MyD88, and NF-κB-mediated luciferase activity was strongly reduced by Pg-EE in a dose-dependent manner (Figure 2B). We assessed the expression level of transcription factor NF-κB subunits, p65 and p50. RAW 264.7 cells were treated with Pg-EE (150 µg/mL), along with LPS, for 5, 15, 30, and 60 min (Figure 2C). The phosphorylation levels of the NF-κB subunits p65 and p50 were decreased with Pg-EE treatment. We can confirm that Pg-EE blocks the NF-κB pathway because the expression levels of phospho-p65 and phospho-p50 decreased.

### 3.3. Regulatory Mechanism of Pg-EE in NF-κB Pathways

**Figure 3:**
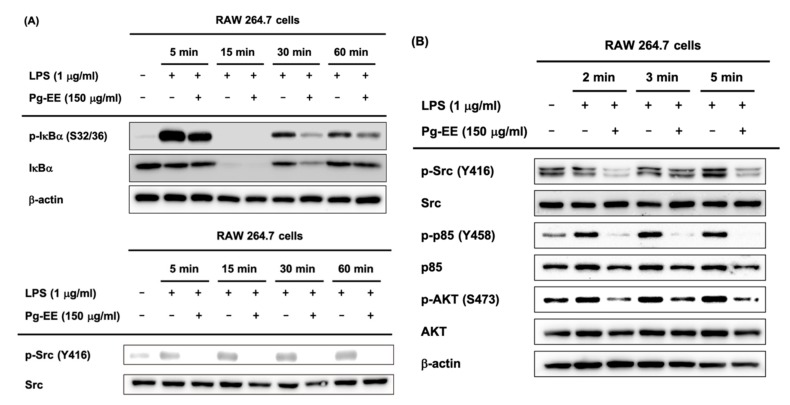
Effect of Pg-EE on the activation of signaling molecules in the NF-κB pathway. (**A**,**B**) Employing western blot analysis, the total and phosphorylated protein levels of inhibitor of kappa B alpha (IκBα), AKT, p85, Src, and β-actin were determined using whole cell lysates from LPS (1 μg/mL)-stimulated RAW264.7 cells with or without Pg-EE (150 μg/mL) in a time-dependent manner. −: no treatment and +: treatment.

To identify which specific protein is targeted by Pg-EE in inhibiting the NF-κB pathway, we analyzed the phosphorylated form of NF-κB-related proteins, including p85, a subunit of PI3K [39], IκBα, AKT, and Src. Phosphorylated IκBα was detected with LPS induction during 5, 15, 30, and 60 min and phosphorylated p85 (Tyr458), AKT (Ser473), and Src (Tyr416) were detected with LPS induction during 2, 3, and 5 min (Figure 3A,B). Src is the most upstream protein of the NF-κB pathway, and in order for the NF-κB signaling to progress, the activation of Src is required in activated macrophages [40]. Src undergoes intermolecular autophosphorylation at tyrosine 416, and its phosphorylation promotes the kinase activity of Src, which in turn leads to the phosphorylation of p85 [41]. Western blot assay data demonstrated that the phosphorylated forms of NF-κB pathway-related proteins, such as Src, were decreased by Pg-EE at short (2, 3, and 5 min) and longer time points (5, 15, 30, and 60 min). Therefore, we can conclude that Src is the target of Pg-EE, and the inhibition of Src kinase results in the reduced activation of proteins downstream of Src.

### 3.4. Anti-Inflammatory Effects of Pg-EE by Targeting Src Kinases

**Figure 4:**
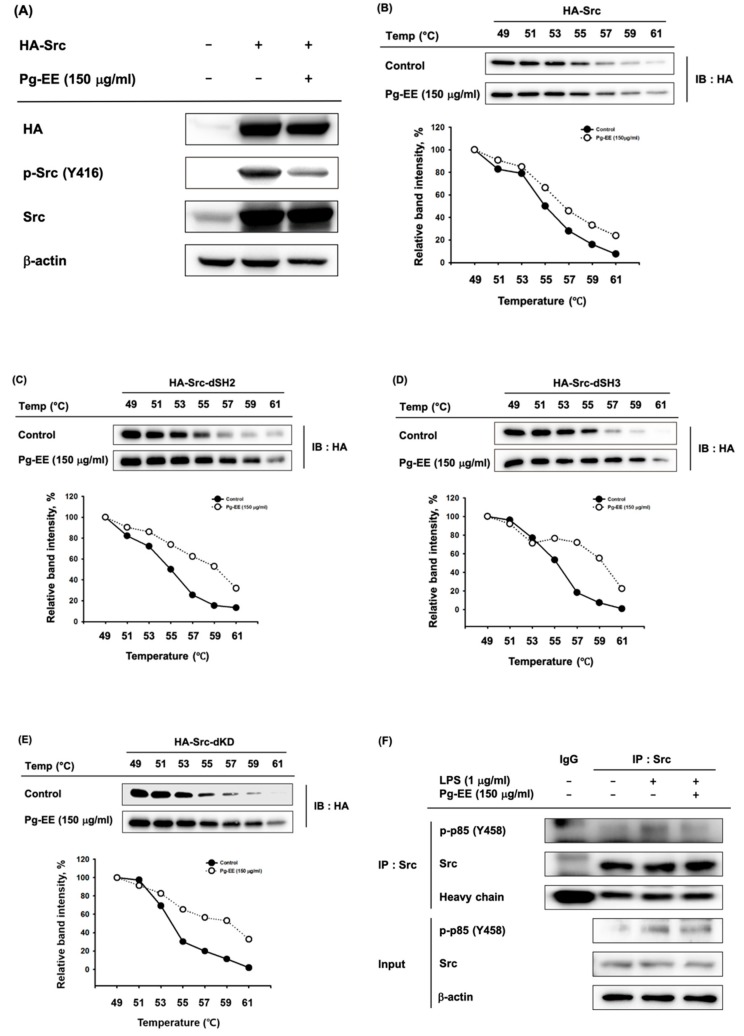
(**A**) HEK293T cells were transfected with HA-Src constructs for 24 h and treated with Pg-EE as indicated. Western blot analysis was performed for HA, total Src, and phosphorylated Src (Tyr416). (**B**–**E**) After overexpressing the Src and Src domain deletion constructs in HEK293T cells, a cellular thermal shift assay (CETSA) was performed with Pg-EE (150 µg/mL) or DMSO (as a control). Stabilization of Pg-EE on Src was examined by western blot analysis. The calculation of band intensity of Src in Pg-EE- and DMSO-treated groups was performed with Image J. (**F**) RAW264.7 cells (5 × 10^6^ cells/mL) were incubated with Pg-EE in the presence or absence of LPS (1 µg/mL) for 0 or 5 min. The binding of phospho-p85 (Tyr458) to Src was detected in whole cell lysates by immunoprecipitation with antibodies to Src and immunoblotting with antibodies to p-p85. −: no treatment and +: treatment.

To confirm that Pg-EE inhibits Src kinase and downstream signaling, we utilized a method of overexpressing the Src kinase. We overexpressed the plasmids expressing HA-Src for 24 h in HEK293T cells and then treated cells with Pg-EE 150 µg/mL for another 24 h. The results showed that p-Src was decreased by treatment with Pg-EE (Figure 4A). To examine the direct interaction of Src and Pg-EE, we used CETSA to evaluate the thermal stabilization of a protein bound to a ligand [42]. Proteins were degraded as the temperature increased to 61 °C, but the degradation level in the Pg-EE-treated group was lower than that in the control group (Figure 4B). These results indicate that the binding of Pg-EE and Src leads to the increased thermostabilization of Src and confirms that Pg-EE binds with Src. We next explored the specific domain of Src to which Pg-EE binds. Src contains an SH4 domain, a unique segment, an SH3 domain, an SH2 domain, and a protein-tyrosine kinase domain [43]. Therefore, we performed CETSA with SH2 domain deletion, SH3 domain deletion, and kinase domain deletion mutants of Src (Figure 4C–E). For all mutant proteins, the degree of degradation of Src with Pg-EE was lower than that of the control group. These results indicate that Pg-EE is bound to domains other than these three domains in Src, and the precise interaction domain should be explored in a follow-up study.

Through interaction of the SH2 domain of Src and p85, Src kinase activity phosphorylated p85 [44]. As shown in Figure 3B, treatment with LPS for 5 min led to the maximum inhibition of phosphorylated Src by Pg-EE, so we used LPS treatment for 5 min in immunoprecipitation analysis. The decreased expression levels of phospho-p85 confirm that the formation of a molecular complex involving p85 and Src was decreased by Pg-EE (Figure 4F).

### 3.5. In Vivo Anti-Inflammatory Effect of Pg-EE on HCl/EtOH-Induced Acute Gastritis

**Figure 5:**
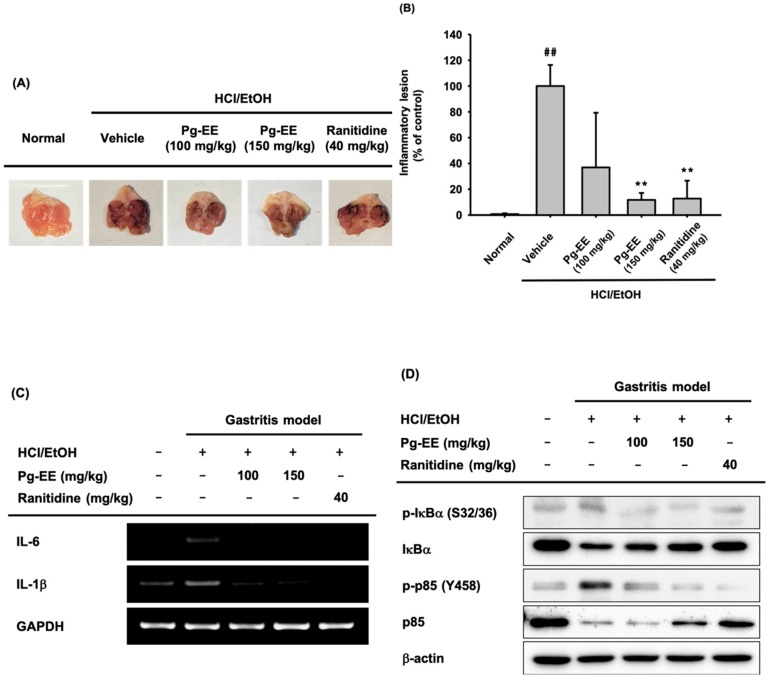
Effect of Pg-EE on the inflammatory symptoms in an HCl/EtOH-induced gastritis model. (**A**) Pg-EE (100 or 150 mg/kg) or ranitidine (40 mg/kg) was orally injected into mice three times a day for two days, and gastritis was induced by HCl/60% EtOH for 1 h before mice were sacrificed. (**B**) Stomach inflammatory lesions were photographed and quantified through ImageJ. (**C**) The mRNA expression levels of IL-6, IL-1β, and GAPDH in the gastritis model treated with Pg-EE (100 mg/kg or 150 mg/kg) or ranitidine (40 mg/kg) were determined using semi-quantitative RT-PCR. (**D**) The protein levels of total and phospho-IκBα (Ser32/36) and -p85 (Tyr458) were detected by an immunoblotting assay. ##: *p* < 0.05 compared to the normal group, *: *p* < 0.05 and **: *p* < 0.01 compared to the control group. −: no treatment and +: treatment.

Finally, to investigate the anti-inflammatory effect of Pg-EE in vivo, we established HCl/EtOH-induced gastritis mice and administered either Pg-EE (100 mg/kg or 150 mg/kg) or ranitidine (40 mg/kg). Pg-EE (150 mg/kg) clearly decreased the inflammatory lesions up to 88%, similar to ranitidine (40 mg/kg), at 87% (Figure 5A,B). We examined the mRNA expression levels and protein levels of NF-κB pathway-related factors in stomach lysates from the treated mice. The mRNA expression levels of IL-6 and IL-1β and the protein levels of phosphorylated IκBα and p85 were decreased by Pg-EE (Figure 5C,D). Therefore, we can conclude that Pg-EE has anti-inflammatory effects by inhibiting progression of the NF-κB pathway both in vitro and in vivo.

## 4. Discussion

The genus *Potentilla* is widely used in Asia, Europe, and North America as a traditional medicine and has shown antioxidant, antimicrobial, anti-inflammatory, and anti-ulcerogenic effects [22,23,24]. However, no study has examined the mechanism of anti-inflammatory activities of *P. glabra* induced by LPS in macrophages. Therefore, this study focused on identifying the factor targeted by Pg-EE in its inhibition of inflammatory mechanisms and demonstrating the anti-inflammatory effect.

When RAW264.7 cells were induced with LPS, Pam3CSK4, or Poly(I:C) and treated with Pg-EE, the released NO production level was decreased (Figure 1A–C). This result indicates that Pg-EE exhibits anti-inflammatory effects without cytotoxicity. Pg-EE clearly suppressed the mRNA expression levels of pro-inflammatory cytokines of iNOS, TNF-α, IL-6, and IL-1β (Figure 2A). A luciferase assay was conducted to determine which pathways were affected, and the results showed that the activation of the transcription factor NF-κB was suppressed by Pg-EE. The western blot assay revealed that phosphorylated subunits of NF-κB, p65, and p50, were also decreased with Pg-EE treatment. Therefore, we found that Pg-EE regulates the NF-κB pathway (Figure 2B,C).

The downregulation of NF-κB signaling plays an important role in suppressing inflammation. Therefore, we examined whether Pg-EE has an effect on the activity of upstream signaling molecules, such as Src, p85, AKT, and IκBα, to identify the direct target. These experiments may also help identify potential therapeutic targets. We found that Pg-EE inhibited the activation of kinase Src, p85, AKT, and IκBα at 5, 15, 30, and 60 min (Figure 3A,B). Src and p85 are the most upstream kinases in the NF-κB signaling pathway. Therefore, we predicted that Src and p85 were rapidly activated with LPS stimulation and confirmed the effect of Pg-EE on their activity at early time points. Pg-EE effectively suppressed the activity of Src and p85 in LPS-stimulated macrophages at 2, 3, and 5 min (Figure 3B), indicating that Pg-EE targets very upstream signaling molecules in the NF-κB signaling pathway in terms of its anti-inflammatory activity.

To evaluate whether Pg-EE targets upstream NF-κB signaling molecules, we used Src-overexpressed HEK293T cells. Pg-EE dramatically suppressed the activity of Src in Src-transfected HEK293T cells (Figure 4A). We next examined whether Src is the target of Pg-EE (Figure 4B–E). We performed CETSA experiments to determine interactions between Pg-EE and Src and also sought to identify which of the various domains of Src interacted with Pg-EE. We evaluated three domains in Src, including the SH2, SH3, and kinase domains, but none interacted with Pg-EE. In our next studies, we plan to identify the precise binding domain. Through the experiments presented in this paper, we have demonstrated that Pg-EE interacts with Src, and further immunoprecipitation inhibited the activation of p85 as a downstream protein of Src (Figure 4F).

Following the in vitro experiments, we examined the anti-inflammatory efficacy of Pg-EE using an HCl/EtOH-induced acute gastritis model in vivo. Pg-EE reduced inflammatory lesions and the incidence of pro-inflammatory cytokines and activation of NF-κB pathway-related proteins in the acute gastritis model (Figure 5). We performed HPLC to identify the components of Pg-EE using standard preparations of quercetin, naringenin, and kaempferol. These flavonoids are polyphenolic compounds that are commonly present in most plants and are well-known to have anti-inflammatory effects [45]. Quercetin represented approximately 90% of the total flavonoids in Pg-EE. Quercetin and kaempferol were reported to reduce iNOS, TNF-α, and IL-6 release from LPS-treated RAW264.7 cells and NF-κB pathway signaling molecules [46,47]. We also confirmed the suppressive effect of quercetin with an NO production assay (Figure 1G,H). These reports are in agreement with our demonstrated effects of Pg-EE on mRNA production, NF-κB luciferase activity, and active forms of NF-κB signaling molecules. These results support the possibility that Pg-EE can be used as an anti-inflammatory medicine component.

## 5. Conclusions

**Figure 6:**
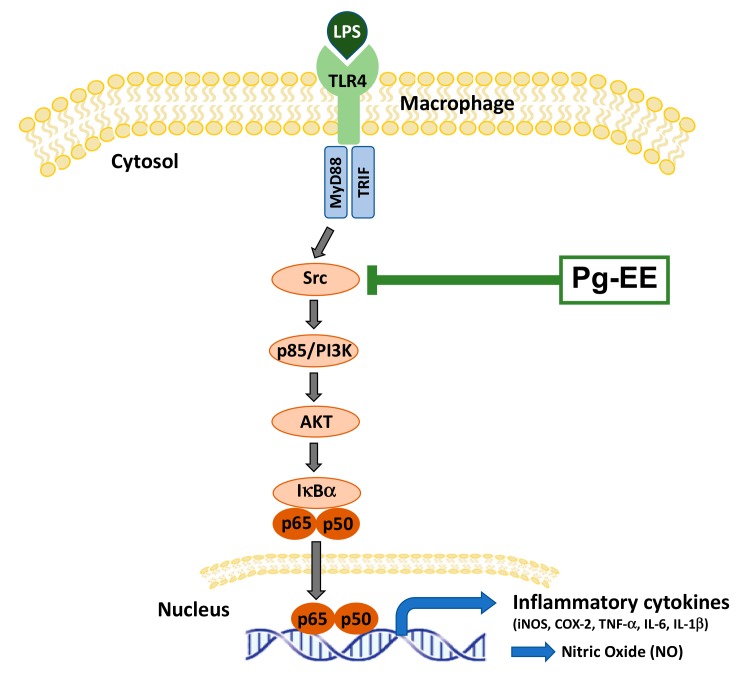
Scheme of the pathway of Pg-EE-mediated anti-inflammatory action. Pg-EE suppresses the phosphorylation of NF-κB pathway components and translocation of p65 and p50 into the nucleus, leading to the inhibition of inflammatory responses. LPS, lipopolysaccharide; TLR4, toll-like receptor 4; MyD88, myeloid differentiation factor 88; TRIF, toll-receptor associated activator of interferon; Pg-EE, *Potentilla glabra* var. mandshurica (Maxim.) Hand.-Mazz. ethanol extract; PI3K, phosphoinositide 3 kinase; IκBα, inhibitor of κBα.

We confirmed that Pg-EE has anti-inflammatory efficacy both in vitro and in vivo. Pg-EE decreased NO production and the mRNA expression levels of pro-inflammatory cytokines. The luciferase assay confirmed that Pg-EE inhibits the activation of NF-κB signaling and the inflammatory process by inhibiting the activation of the Src kinase in vitro (Figure 6). In addition, the administration of Pg-EE to HCl/EtOH-induced acute gastritis mice resulted in reduced gastritis lesions and decreased pro-inflammatory cytokines and NF-κB pathway-related proteins. As a result, this research confirmed the anti-inflammatory efficacy of Pg-EE at the mechanism level, and also raises the possibility that Pg-EE can be applied as a therapeutic agent. Nonetheless, several additional works regarding activity-guided fractionation to identify active components from Pg-EE; finding other molecular targets, such as PI3K or AKT, in both the NF-κB pathway and other inflammatory signaling cascades, such as the AP-1 pathway; testing the inhibitory effect of Pg-EE in other inflammatory cells, such as tissue epithelial cells; and evaluating the therapeutic activity of this extract with other inflammation models, such as a chronic inflammatory disease model (e.g., osteoarthritis model), will be performed in future projects.

## Figures and Tables

**Table 1:** Sequences of primers used in RT-PCR analysis.

Gene		Sequences (5′ to 3′)
iNOS	Forward	5′-CCCTTCCGAAGTTTCTGGCAGCAG −3′
	Reverse	5′-GGCTGTCAGAGCCTCGTGGCTTTGG −3′
TNF-α	Forward	5′-TTGACCTCAGCGCTGAGTTG −3′
	Reverse	5′-CCTGTAGCCCACGTCGTAGC −3′
IL-6	Forward	5′-GGAAATCGTGGAAATGAG −3′
	Reverse	5′-GCTTAGGCATAACGCACT −3′
IL-1β	Forward	5′-CAGGATGAGGACATGAGCACC −3′
	Reverse	5′-CTCTGCAGACTCAAACTCCAC −3′
GAPDH (Mouse)	Forward	5′-CACTCACGGCAAATTCAACGGCAC −3′
	Reverse	5′-GACTCCACGACATACTCAGCAC −3′

## Abbreviations/Nomenclature

Activator protein 1	AP-1
Cyclooxygenase-2	COX-2
Inducible NO synthase	iNOS
Inhibitor of kappa B alpha	IκBα
Inhibitor of kappa B kinase	IKK
Interleukin	IL
Interleukin	IL
Lipopolysaccharide	LPS
Myeloid differentiation primary response 88	MYD88
Nitric oxide	NO
Nuclear factor kappa-light-chain-enhancer of activated B cells	NF-κB
Pathogen-associated molecular patterns	PAMPs
Pattern recognition receptors	PRRs
Phosphoinositide-3-kinase p85	PI3K p85
*Potentilla glabra* Var. mandshurica (Maxim.) Hand.-Mazz ethanol extract	Pg-EE
Spleen tyrosine kinase	Syk
TIR-domain-containing adapter-inducing interferon-β	TRIF
Toll-like receptors	TLRs
Tumor necrosis factor-alpha	TNF-α
